# The effects of everyday-life social interactions on anxiety-related autonomic responses differ between men and women

**DOI:** 10.1038/s41598-023-36118-z

**Published:** 2023-06-12

**Authors:** Marthe Gründahl, Martin Weiß, Kilian Stenzel, Jürgen Deckert, Grit Hein

**Affiliations:** Translational Social Neuroscience Unit, Department of Psychiatry, Psychosomatic and Psychotherapy, Center of Mental Health, University of Würzburg, Margarete-Höppel-Platz 1, 97080 Würzburg, Germany

**Keywords:** Physiology, Psychology

## Abstract

Social buffering, a phenomenon where social presence can reduce anxiety and fear-related autonomic responses, has been studied in numerous laboratory settings. The results suggest that the familiarity of the interaction partner influences social buffering while also providing some evidence for gender effects. In the laboratory, however, it is difficult to mimic the complexity of real-life social interactions. Consequently, the social modulation of anxiety and related autonomic responses in everyday life remains poorly understood. We used smartphone-based Ecological Momentary Assessment (EMA) combined with wearable electrocardiogram sensors to investigate how everyday-life social interactions affect state anxiety and related cardiac changes in women and men. On five consecutive days, 96 healthy young participants (53% women) answered up to six EMA surveys per day, indicating characteristics of their most recent social interaction and the respective interaction partner(s). In women, our results showed lower heart rate in the presence of a male interaction partner. Men showed the same effect with female interaction partners. Moreover, only women showed decreased heart rate and increased heart rate variability with increasing interaction partner familiarity. These findings specify the conditions under which social interactions reduce anxiety-related responses in women and men.

## Introduction

Animal and human studies have shown that the mere presence of a conspecific can reduce physiological stress and fear responses^1,2^, a phenomenon called social buffering^3,4^. So far, social buffering has mostly been investigated in the laboratory. In human studies, participants typically experience a stress or anxiety-inducing situation (e.g., pain induction, public speech) alone or in the presence of another person. Some studies extend the social aspect, for instance by modulating the familiarity (e.g., stranger versus friend) or affiliative behaviour (e.g., social support versus no support) of the interaction partner^1,5^. Social buffering effects are inferred from reductions of reported anxiety or adaptive changes in autonomic responses caused by the interaction partner’s presence or behaviour^6,7^. For example, the presence of social support has been linked to reduced heart rate (HR)^8^, indicating lower acute (social) stress^9,10^. In contrast, a lack of social contact (i.e., social isolation) has been associated with lower heart rate variability (HRV)^11^, reflecting diminished behavioural and emotion-regulatory adaptability^12,13^.

The social buffering effects observed in the laboratory were modulated by various factors, including the gender of the participant as well as the familiarity and gender of their interaction partner. Regarding familiarity, findings suggest stronger social buffering effects in the presence of more familiar interaction partners^14,15^. Regarding the participant’s gender, previous studies imply that men and women experience certain social situations differently. In general, self-reported anxiety tends to be higher in women^16,17^ which is paralleled by stronger physiological reactions such as higher HR^18,19^ and lower HRV^20^ in anxiety-inducing situations. Findings concerning gender effects in social buffering of anxiety are mixed, however. Some studies reported stronger effects in women compared to men^3,21^, while other studies showed the opposite^22,23^. Yet other studies found no gender differences in general anxiety levels^24,25^ or social buffering effects^26,27^.

Regarding the gender of the interaction partner, both men and women showed reduced autonomic responses (systolic blood pressure [BP]) when receiving social support from female but not male social partners^19^. Similarly, other laboratory settings with anxiety-related autonomic measurements revealed social buffering effects in female but not male dyads, e.g., on skin conductance responses^3^. Challenging this result, other studies reported reduced cardiovascular reactivity (systolic BP, HR) in women in the presence of their romantic partner^28^ or when receiving social support from a male friend^29^. Yet another study showed reduced cortisol levels in men in the presence of their (female) romantic partner, but not in women in the presence of their (male) romantic partner^22^. Finally, a meta-analysis revealed no gender differences in the effect of social stress induction on HRV. In both genders, dyadic tasks designed to elicit negative affect decreased HRV, whereas dyadic tasks eliciting positive or no particular valence had no significant influence^26^.

Overall, findings from previous laboratory studies suggest that social presence and contact, particularly with more familiar persons, can reduce anxiety on the subjective and autonomic level, and that the gender of the participants and the interaction partner might play a role. However, the findings are inconsistent and were mainly observed in the laboratory. Capturing the complexity of everyday-life social interactions is difficult in the laboratory, for example, regarding the inclusion of all possible combinations of same- and mixed-gender interaction partners at varying quantities and familiarity levels. Thus, it remains unclear whether social buffering effects found in the laboratory transfer to social situations in everyday life. Here, we used smartphone-based Ecological Momentary Assessment (EMA) with wearable electrocardiogram (ECG) sensors to investigate how everyday-life social interactions affect subjective state anxiety and related cardiovascular changes depending on characteristics of both the participants and their interaction partners. EMA encompasses repeated, real-time subjective and/or physiological assessments of people’s behaviour and experiences in a naturalistic setting. It can integrate multiple within- and between-subject variables simultaneously, thereby further minimizing biases and measurement restrictions associated with laboratory settings. As such, EMA facilitates the economic testing of laboratory findings’ ecological validity in everyday life^30–32^.

Previous studies successfully applied EMA to investigate changes of autonomic responses and started to uncover the effects of important social factors like familiarity in everyday life. In accordance with decreased state anxiety in response to social support from familiar versus unfamiliar persons observed during psychosocial stress in the laboratory^33^, an EMA study revealed lower self-reported anxiety levels when spending time with familiar versus less familiar social partners, particularly in socially anxious individuals^34^. Similar relations were found between interaction partner familiarity and state social interaction anxiety^32^, an anxiety measure closely related to higher state anxiety levels^35^. Other EMA research has revealed reduced cardiovascular responses (i.e., ambulatory BP) in healthy participants when interacting with family members or romantic partners compared to unfamiliar partners^36^, and increased HRV with familiar interaction partners (family, romantic partner) compared to strangers in shy individuals^37^. These studies provided first insights into the effect of social factors like familiarity in everyday-life settings but did not consider the gender of the participants and interaction partners. Extending previous EMA work, we designed an EMA study to investigate how the gender of the participant and the interaction partner affect social buffering of state anxiety and related autonomic responses in everyday life, particularly regarding the effect of familiarity.

We hypothesized that participants would benefit from more familiar interaction partners which would be represented in decreased state anxiety^32^ and HR as well as increased HRV^36,37^, and tested how these effects were influenced by the gender of the participants and their interaction partners. We assumed that women would show higher HR and lower HRV than men during everyday-life social interactions^18,20^. However, based on previous findings^3,19,22^, we additionally hypothesized that men and women would show more adaptive subjective and cardiovascular responses (i.e., lower state anxiety, lower HR, higher HRV) in the presence of female interaction partners. Alternatively, we expected opposite-gender social buffering effects^28,29^ or no effects of the interaction partners’ gender^26^.

## Methods

### Participants

122 healthy men and women aged 18 to 35 years participated in the study (two dropouts)*.* After data curation, the final sample consisted of 96 participants (53.1% female; mean age = 25.17, *SD* = 4.12) with a total of 1 536 observations. Exclusion criteria included current pregnancy or lactation period, cardiovascular illnesses, chronic neurological disorders, acute psychiatric disorders, other severe medical illnesses, psychotropic medication, and visual and motoric impairments. We excluded nine participants after visual inspection of ECG data due to frequent artefacts and 15 participants with invalid baseline measurements. Invalid data were mainly caused by insufficient quality of ECG chest belts. A sensitivity analysis using G*Power software (version 3.1.9.7)^38^ revealed that the final sample size of *N* = 96 had a statistical power of > 0.80 with a 5% type I error rate to detect small to medium effect sizes (*f*^2^ = 0.08) in a multiple regression model with 15 predictors (comparable to our models, see below). Given that the within-subject effects have larger level-1 sample sizes (equivalent to the number of observations nested within persons), we expected to have sufficient power to detect small effects. All participants provided written informed consent. The study protocol was approved by the ethics committee of the medical faculty of the University of Würzburg (vote #180/20) and complies with the Declaration of Helsinki.

### Procedure

**Figure 1:**
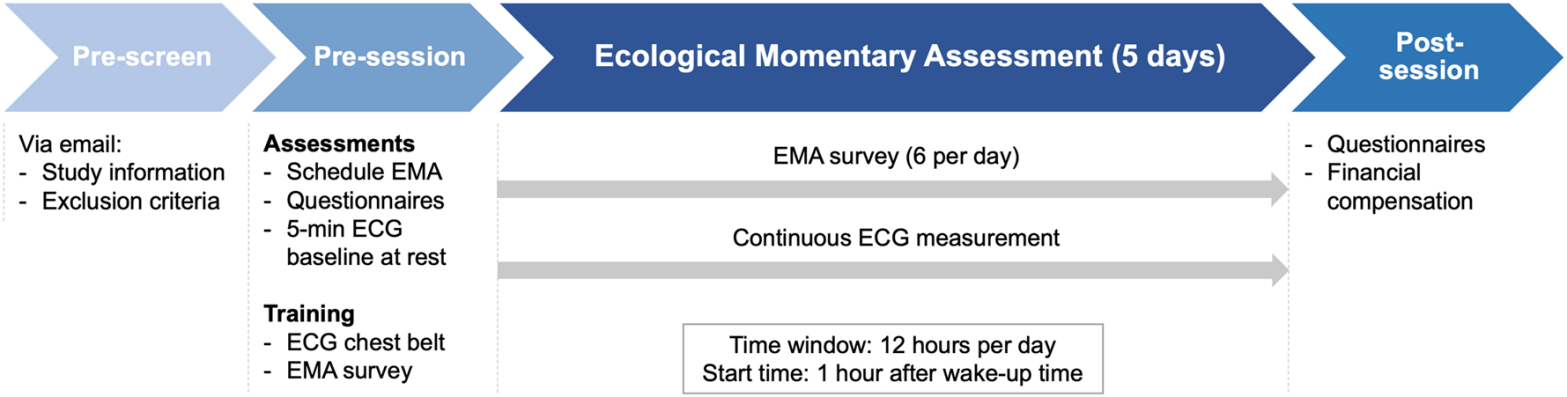
Study procedure. ECG, Electrocardiogram; EMA, Ecological Momentary Assessment.

Participants were invited to a pre-session. A chest belt with ECG sensor was attached and the participant’s schedule for the five EMA measurement days was enquired to adjust the 12-h measurement time windows, starting one hour after the usual wake-up time. Participants filled in sociodemographic and clinical questionnaires. During the subsequent 5-min baseline measurement, the participants sat upright facing a 16:9 computer screen at approx. 140 cm distance. They were asked to relax while watching an audio-visual clip of an aquarium. Afterwards, the participants practiced the correct use of the ECG belt and the EMA questionnaire application (app) delivered on a study smartphone. Supervised by the experimenter, they answered the EMA survey in two imaginary situations (social, non-social) before receiving the study material. On five consecutive days, participants carried the smartphone with them while wearing the ambulatory ECG sensor within the 12-h time windows. Within three days after the final assessment, they returned to the laboratory, filled in questionnaires, and received a financial compensation. An overview of the study procedure is displayed in Fig. 1.

### Measures

#### Trait questionnaires

Participants were screened for depression using the 15-item short form of the Center for Epidemiologic Studies Depression Scale (German: “Allgemeine Depressionsskala—Kurzform”, ADS-K)^39^. In addition, we assessed participants’ trait anxiety and social interaction anxiety using the trait anxiety subscale of the state-trait anxiety inventory (STAI)^40^ and the social interaction anxiety scale (SIAS)^41^, respectively.

#### EMA survey items

EMA surveys were delivered with the movisensXS app (Movisens GmbH) on an android smartphone. State anxiety was assessed with a 10-item short form of the STAI state anxiety subscale^42^ rated on a Likert scale from 1 (“not at all”) to 8 (“totally”). Participants indicated the time of their most recent social interaction (“now”, “within the last 30 min”, “more than 30 min ago”). For social interactions ≤ 30 min ago, they answered the social interaction questionnaire (for a detailed item list, see Table S1). Otherwise, they answered a similarly structured activity questionnaire to prevent avoidance behaviour due to time savings (see Table S2). The social interaction questionnaire first assessed the type (“direct contact”, “telephone call”, “e-mail/letter”, “SMS”, “social media”; “private”, “job-related”), the approximate start and duration of the social interaction (slider scales with three anchors: “ < 1 min”, “15 min”, “ > 30 min”), and the quantity (“1” to “5 or more”) and gender (“female”, “male”, “mixed”) of the interaction partners. The familiarity of the interaction partners (“I know the other person/one of the other persons well.”) was assessed continuously with a Likert scale ranging from 1 (“not at all”) to 8 (“very”)^43,44^, given that the categorical measurement of familiarity in previous studies may overlook relations like estranged family members or familiar long-time colleagues. State social interaction anxiety was averaged from two items (“I worried about what the other person(s) thought of me”, “I was worried that I would say or do the wrong things”) rated on a Likert scale from 1 (“not at all”) to 9 ("very")^45,46^. We assessed the pleasantness of the interaction (“How (un)pleasant was the interaction?”) on an 8-point Likert scale ranging from 1 (“very unpleasant”) to 8 (“very pleasant”)^47^. The final screen assessed ECG control variables (e.g., consumption of caffeine/nicotine/alcohol during the last hour)^48,49^.

#### Autonomic measures

HR and HRV were measured continuously with an ambulatory ECG, i.e., the movisens EcgMove4 sensor (Movisens GmbH, Karlsruhe, Germany). HR describes the number of heartbeats in beats per minute (bpm). In the presence of a stressor, HR is increased by vagal withdrawal and sympathetic activation^9,12^. HRV is a quantitative marker of autonomic nervous system activity influenced by both sympathetic and parasympathetic activity^50,51^. It describes the fluctuation in the time intervals between adjacent heartbeats, which show less linearity and more spatial and temporal complexity in healthy individuals (i.e., higher HRV)^52,53^. Higher HRV relates to higher skills, engagement, and stress regulation in social situations^13,54^, including the adaptive regulation of HR^55^. This study’s HRV measure is the root mean square of successive differences between heartbeats (RMSSD) measured in ms. RMSSD estimates vagal cardiac control and reflects short-term parasympathetic variations. There is support that time-domain HRV measures like RMSSD show higher robustness to (motion) artefacts than frequency-domain HRV measures^56^. RMSSD is an appropriate measure for long- and short-term time windows^57^ and EMA research^58,59^. For comprehensive guidelines, see^50^ or^48^.

The ECG sensor was attached to a chest belt worn at the base of the sternum with direct skin contact of two dry electrodes. Participants were instructed to clean the skin beneath the electrodes with alcohol pads (70% isopropyl alcohol) prior to attachment. The sensor collected single channel ECG data (resolution = 12 bits, sampling rate = 1 204 Hz). Participants were asked not to consume a heavy meal, coffee, alcohol or tobacco, and to only drink low amounts of water in the two hours prior to the pre-session^48^. All baseline ECG measurements were collected between 12 and 6 pm.

### Data analysis

#### EMA survey data curation

We screened the EMA survey data for invalid or incoherent data. Continuous within-person variables were person-centred and continuous between-person variables were grand mean-centred^60^. We categorized the social interaction type into “direct” or “virtual” and transformed consumption indications into binary variables (“yes”, “no”). Finally, we added a binary variable for the interaction partner quantity (“1”, “2 or more”) to account for the constitutional absence of mixed-gender social interactions with one person only.

#### ECG data curation and heart rate variability calculation

After visual inspection of ECG data, we applied the movisens DataAnalyzer software (version 1.13.8) to convert the ECG signals into HRV indices. The software uses an automated algorithm to detect artefacts. Its output is based on minute-by-minute calculations derived from 2-min segments. The DataAnalyzer detects R-peaks^61^, marks signal amplitude and number of zero crossings per s outside a normal physiological range as artefacts, and filters invalid R-peaks and artefacts^62^ (for more details, see supplement). Bodily movement (sampling rate = 64 Hz) was transformed into 1-min epochs.

ECG and EMA data were aligned and imported into R (version 4.2.0). We rounded ECG data to whole-minute values, excluded ECG segments labelled as invalid, and calculated mean scores for HR and RMSSD at baseline (first minute excluded). We calculated separate outlier detections for baseline and EMA values of HR and RMSSD. Values outside the upper and lower quartile by at least 1.5 times the interquartile range were removed^63^. 73.4% of rows had valid HR and RMSSD. We extracted mean HR and RMSSD values for each social interaction (time retrieved from slider scales, transformed to 1-min-intervals; first and last segment of time window excluded). The survey prompt marked the end of ongoing social interactions. We removed social interactions shorter than 60 s. A natural logarithmic transformation was performed to correct for skewness (RMSSD). The final 96 participants provided a total of 1 536 valid observations, i.e., 16 social interaction assessments on average (*SD* = 5.08, range = 5–27).

#### Statistical analysis

To test our hypotheses, we calculated three linear mixed models with participant as random intercept and state anxiety, HR, and RMSSD as dependent variables, respectively, using the ‘lme4’ package^64^. Differences in main variables between male and female participants were calculated using *t*-tests. All linear mixed models were tested against basic models without interactions. Model significance was calculated with the ‘lmerTest’ package^65^, applying Satterthwaite’s method to estimate degrees of freedom and *p*-values. We assessed multicollinearity within the models by calculating variance inflation factors (VIF) with the ‘performance’ package^66^. The ‘sjPlot’ package provided ICC and R^2^^67^. We conducted simple slope analyses for significant interactions with continuous variables using the ‘interactions’ package^68^ and post hoc comparisons using pairwise *t*-tests (Bonferroni-corrected) for interactions with categorical variables.

First, as a manipulation check for the expected relationship between subjective anxiety and autonomic responses, we created two simple models with state anxiety as outcome and HR or RMSSD during EMA as predictor. We added essential autonomic control variables (caffeine, nicotine, alcohol, acceleration, baseline; see below).

Second, we created the main models. The state anxiety, HR, and RMSSD models included the predictors participant’s gender (gender participant; two levels: female, male), familiarity (familiarity IP; centred within [cw]) and gender of the interaction partner (gender IP; three levels: female, male, mixed; reference: female), state social interaction anxiety (state SI anxiety; cw), trait social interaction anxiety (SIAS; centred between [cb]), and social interaction type (SI type; two levels: direct, virtual). HR and RMSSD are sensitive to a number of potential confounds^48,49,56^. To enhance model comparability, we therefore included the following covariates into all three models: duration of the social interaction (SI duration; measured in s, cw), interaction partner quantity (quantity IP; two levels: 1, 2 or more), and consumption of caffeine, nicotine, and alcohol within the last hour (two levels each: yes, no). For HR and RMSSD, we additionally included movement acceleration (accel; mean, cw) as an indicator of physical activity, a factor which can enhance HR^49^ and interfere with vagal cardiac control as reflected in HRV^69^. To account for individual differences in autonomic baseline levels, the HR and RMSSD models further included the covariate HR at baseline (cb) and RMSSD at baseline (cb), respectively, as recommended for ECG assessments^48^. All three models originally included two interactions: gender participant x gender IP, and gender participant x familiarity IP. For state anxiety, the interaction effects were non-significant, and model comparison with likelihood ratio tests indicated no difference in model fit with or without the interactions (*p* = 0.734). Therefore, we chose the basic state anxiety model as main model. For the HR and RMSSD models, model comparison indicated better model fit of the interaction models (both *p*s’ < 0.001), which were therefore chosen as main HR and RMSSD models.

Finally, we compared the main models to more complex (and less parsimonious) models. First, we added age (cb) and depression scores (ADS-K; cb) as additional control variables. Second, we integrated social interactions with romantic partners (two levels: yes, no) as potential confounding variable for gender IP effects. Third, we tested two additional control interactions (SI type x accel, gender IP x quantity IP) for the autonomic models. Age and ADS-K improved model fit for the anxiety model and were therefore added to the final anxiety model. There were no other significant improvements in model fit (all *p*s' > 0.05) and thus no other changes. Note that we also conducted exploratory analyses to test for moderating effects of social interaction pleasantness on familiarity IP^47,70^ and of SIAS on familiarity IP^41^ and SI type^71^. The interactions remained non-significant in the state anxiety model (all *p*s’ > 0.484). For HR and RMSSD, the interactions also remained non-significant, with the interaction between pleasantness and familiarity showing a trend towards significance for HR (β = 0.15, *SE* = 0.08, *p* = 0.056) and RMSSD (β = − 0.01, SE = 0.00, *p* = 0.074; all other *p*s’ > 0.621). However, the inclusion of the interactions did not improve model fit (HR: *p* = 0.393; RMSSD: *p* = 0.484) and was therefore discarded.

##### Ethical standards

The authors assert that all procedures contributing to this work comply with the ethical standards of the relevant national and institutional committees on human experimentation and with the Helsinki Declaration of 1975, as revised in 2008.

## Results

### Sample characteristics

The observations consisted of 78.4% direct and 21.6% virtual social interactions. 40.2% of social interactions took place with female, 29.5% with male, and 30.3% with mixed interaction partners. Overall, 42.5% of social interactions included two or more interaction partners (16.9% female, 12.1% male, 71.0% mixed). 57.5% of one-person social interactions were with female interaction partners (see Supplementary Table S5 for additional characteristics of social interactions). Sample characteristics and gender differences are reported in Table 1 (see Table S6 for correlations between HR, RMSSD, and trait questionnaires). Compared to men, women showed higher mean levels of HR during social interactions. Women also tended to show higher HR during baseline and higher depression scores (for numeric values, see Table 1).

**Table 1:** Characteristics of the total sample and the female and male participants.

	Total sample (*N* = 96)		Female subsample (*n* = 51)		Male subsample (*n* = 45)				
	*M*	*SD*	*M*	*SD*	*M*	*SD*	Sample comparison	*p*	Cohen’s* d*
Age	25.17	4.12	24.55	4.06	25.87	4.12	*t*(94) = 1.58	.119	.322
BMI	23.13	3.62	21.93	3.62	24.50	3.12	*t*(94) = 3.71	< .001	.762
HR (baseline)	71.76	11.04	73.53	10.47	69.74	11.44	*t*(94) = − 1.70	.093	.346
RMSSD (baseline)	47.52	23.79	48.69	25.29	46.18	22.16	*t*(94) = − 0.51	.609	.105
HR (SI)	82.95	7.97	84.72	7.42	80.94	8.17	*t*(94) = -2.38	.019	.485
RMSSD (SI)	35.90	9.35	35.89	9.61	35.91	9.15	*t*(94) = 0.01	.991	.002
State anxiety	26.09	10.95	26.69	10.84	25.40	11.16	*t*(94) = − 0.57	.568	.117
State SI anxiety	2.59	1.31	2.61	1.38	2.57	1.23	*t*(94) = − 0.16	.876	.032
SIAS	20.41	10.85	20.04	11.04	36.62	9.61	*t*(94) = .35	.726	.072
ADS-K	10.14	4.17	10.88	4.44	9.29	3.70	*t*(94) = − 1.90	.051	.390
STAI-Trait	37.16	10.12	37.63	10.63	36.62	9.61	*t*(94) = − 0.48	.630	.099

ADS-K, Allgemeine Depressionsskala; BMI, Body mass index; HR, Heart rate; RMSSD, Root mean square of successive differences between heartbeats; SI, Social interaction; SIAS, Social Interaction Anxiety Scale; STAI, State Trait Anxiety Inventory; state SI anxiety, State social interaction anxiety.

### State anxiety

The manipulation check confirmed a relationship between subjective and autonomic indicators of anxiety. Higher state anxiety correlated with higher HR during social interactions (β = 0.17, *p* < 0.001) but not with changes in RMSSD (β = − 1.20, *p* < 0.184).

**Figure 2:**
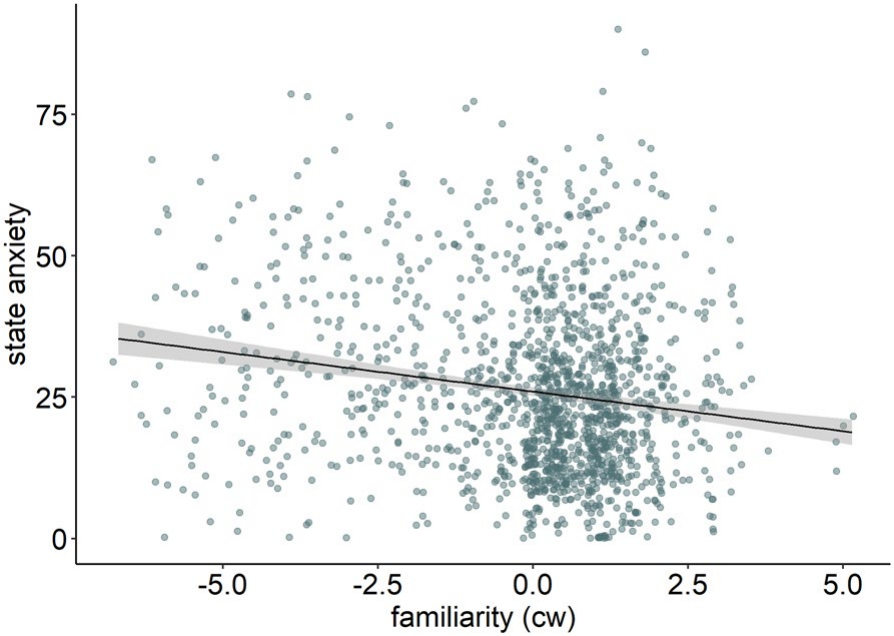
Main effect of familiarity (centred within, cw) on state anxiety. Shaded errors indicate 95% confidence intervals.

Details of the results for the state anxiety model are presented in Table 2 (left column). VIF (all ≤ 2.39) were in an acceptable range^72^. There was a main effect of familiarity IP (β = − 0.94, *p* < 0.001; see Fig. 2), indicating lower state anxiety after interacting with more familiar interaction partners.

**Table 2:** Linear mixed models examining the association of social interaction variables and participant variables with heart rate (HR), heart rate variability (RMSSD [ln]), and state anxiety.

Models	State anxiety			HR			RMSSD (ln)		
Fixed effects	est. (*SE*)	*t*	*p*	est. (*SE*)	*t*	*p*	est. (*SE*)	*t*	*p*
(Intercept)	13.41 (2.79)	4.80	< .001	79.69 (0.98)	81.70	< .001	3.54 (0.04)	86.35	< .001
Within-person effects									
Familiarity IP^a^	− 0.94 (0.16)	− 5.99	< .001	0.24 (0.17)	1.40	.163	− 0.03 (0.01)	− 4.06	< .001
Gender IP (f-m)	0.80 (0.79)	1.02	.308	2.13 (0.84)	2.53	.012	− 0.05 (0.04)	− 1.26	.207
Gender IP (f-mix)	0.62 (1.07)	0.58	.562	2.83 (0.93)	3.04	.002	0.02 (0.04)	0.59	.553
State SI anxiety^a^	1.96 (0.22)	8.87	< .001	0.49 (0.16)	3.09	.002	− 0.01 (0.01)	− 1.96	.050
Quantity IP (1-2 or more)	− 0.63 (0.96)	− 0.66	.508	0.97 (0.69)	1.41	.159	− 0.08 (0.03)	− 2.79	.005
SI type (direct-virtual)	2.34 (0.80)	2.94	.003	− 2.57 (0.58)	− 4.46	< .001	0.07 (0.02)	2.94	.003
SI duration^a^	− 0.00 (0.00)	− 2.42	.015	− 0.00 (0.00)	− 1.40	.161	0.00 (0.00)	2.06	.040
Caffeine (yes–no)	− 0.55 (0.80)	− 0.68	.494	− 0.56 (0.57)	− 0.99	.325	0.04 (0.02)	1.77	.076
Nicotine (yes–no)	0.72 (1.98)	0.36	.716	6.73 (1.40)	4.81	< .001	− 0.21 (0.06)	− 3.49	< .001
Alcohol (yes–no)	− 1.89 (1.28)	− 1.48	.140	4.58 (0.91)	5.01	< .001	− 0.11 (0.04)	− 2.81	.005
Accel^a^				121.88 (4.15)	29.39	< .001	− 3.05 (0.18)	− 17.20	< .001
Between-person effects									
Gender participant (f-m)	0.14 (1.96)	0.07	.944	5.44 (1.34)	4.08	< .001	− 0.10 (0.06)	− 1.81	.071
SIAS^b^	0.23 (0.09)	2.46	.014	0.07 (0.06)	1.27	.205	− 0.00 (0.00)	− 1.49	.136
HR (baseline)^b^				0.43 (0.05)	7.78	< .001			
RMSSD (baseline)^b^							0.25 (0.05)	5.20	< .001
ADS-K^2^	1.20 (0.25)	4.72	< .001						
Age^2^	0.56 (0.24)	2.30	.021						
Interactions									
Familiarity IP^a^ x gender participant (f-m)				− 0.78 (0.22)	− 3.55	< .001	0.05 (0.01)	5.10	< .001
Gender participant (f-m) x gender IP (f-m)				− 4.72 (1.16)	− 4.07	< .001	0.15 (0.05)	3.08	.002
Gender participant (f-m) x gender IP (f-mix)				− 3.08 (1.05)	− 2.92	.004	0.05 (0.05)	1.16	.246
ICC	0.39			0.33			0.32		
Marginal R^2^	0.20			0.44			0.23		

^a^centred within; ^b^centred between. accel, Acceleration; ADS-K, Allgemeine Depressionsskala; f, Female; IP, Interaction partner; HR, Heart rate; m, Male; mix, Mixed genders; RMSSD, Root mean square of successive differences between heartbeats; SI, Social interaction; SIAS, Social Interaction Anxiety Scale; state SI anxiety, State social interaction anxiety.

In addition, lower state anxiety was related to lower state SI anxiety (β = 1.96, *p* < 0.001), and state anxiety was lower during direct compared to virtual social interactions (χ^2^(1) = 8.65, *p* = 0.003). On the between-person level, state anxiety increased with age (β = 0.23, *p* = 0.014), trait SI anxiety (β = 0.56, *p* = 0.021), and depression scores (β = 1.20, *p* < 0.001). There were no significant gender effects.

### Heart rate

**Figure 3:**
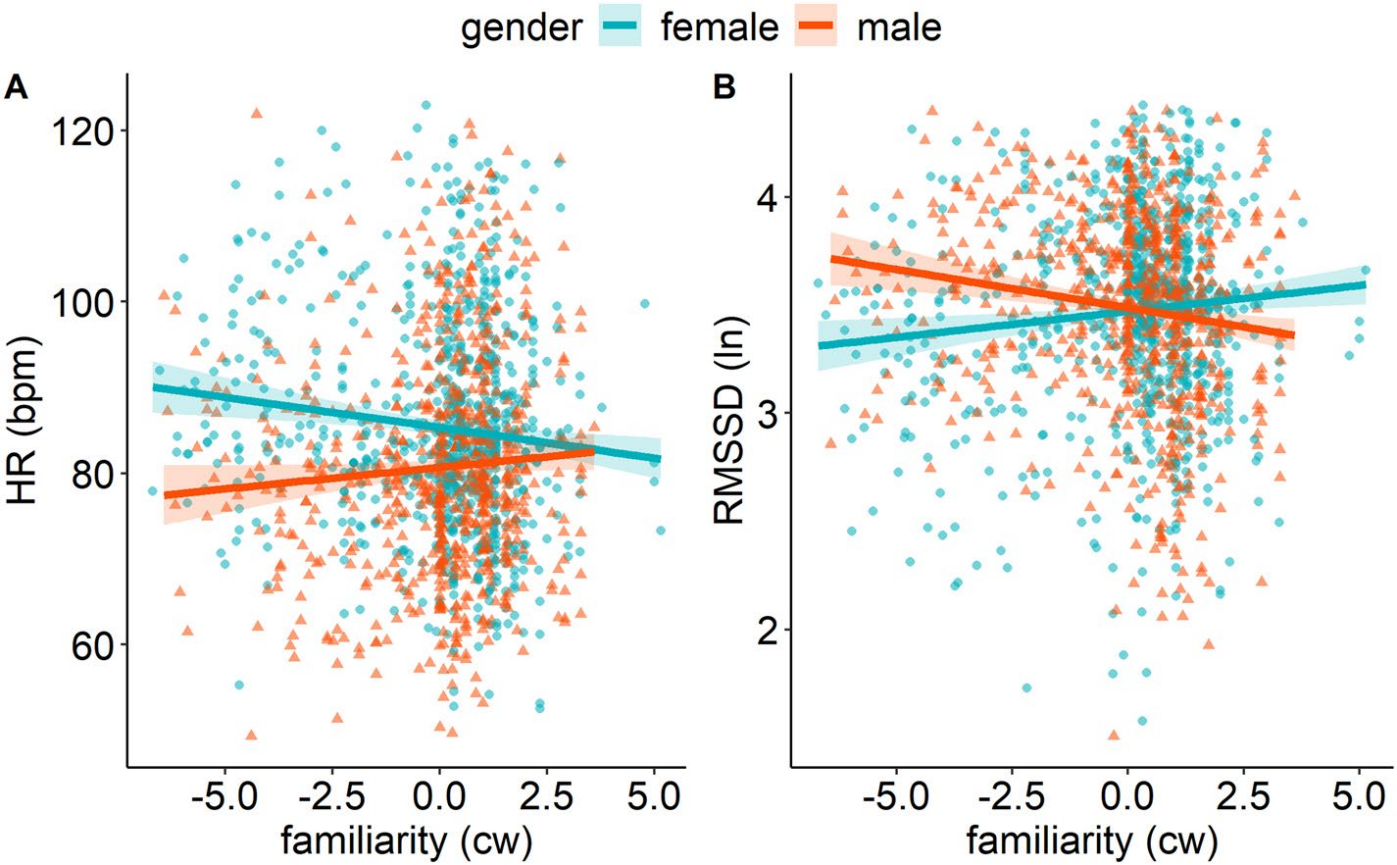
Prediction of HR (**A**) and RMSSD (ln; **B**) by familiarity x gender participant, i.e., in female (turquoise) and male (red) participants. Shaded errors indicate 95% confidence intervals. bpm, Beats per min; cw, Centred within.

Details of the results for the HR model are presented in Table 2 (middle column). VIF (all ≤ 7.45) were in an acceptable range^72^. The HR results showed a significant main effect of state SI anxiety (β = 0.49, *p* = 0.002), representing higher HR with higher state social interaction anxiety. In addition, the main effect of SI type (χ^2^(1) = 19.92, *p* < 0.001) indicated lower HR during virtual versus direct social interactions. The main effect of the participant’s gender (χ^2^(1) = 16.61, *p* < 0.001) reflected higher HR in women. The participant’s gender also interacted with familiarity IP (χ^2^(1) = 12.60, *p* < 0.001; Fig. 3A). Follow-up analyses for the simple slopes of familiarity for female and male participants revealed a negative slope for women (β = − 0.54, *p* < 0.001) but no effect for men (β = 0.24, *p* = 0.163).

**Figure 4:**
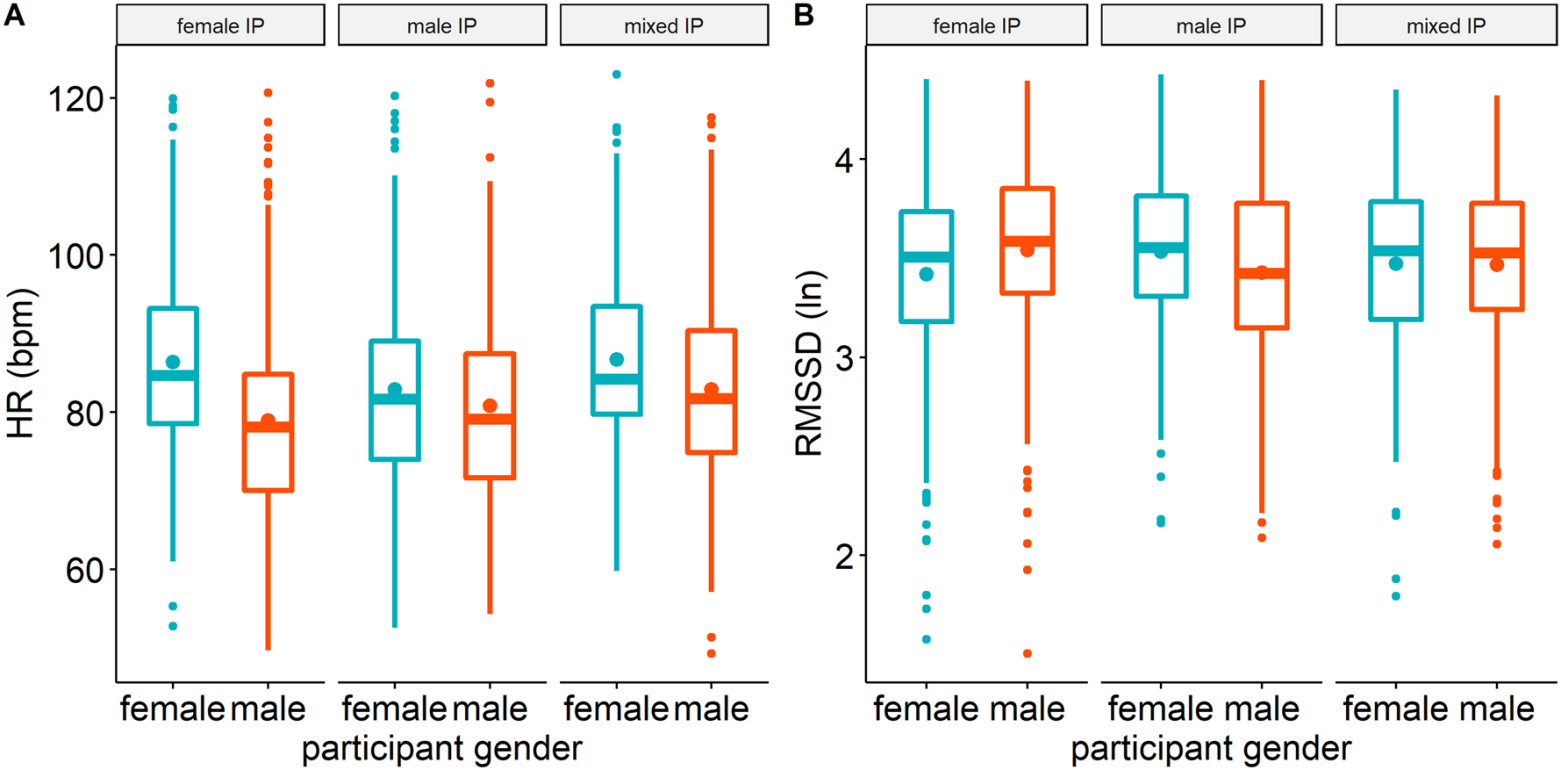
HR (**A**) and RMSSD (**B**) in female (turquoise) and male (red) participants in social interactions with female, male, and mixed interaction partners (IP). Dots within boxplots represent group mean, lines represent medians. bpm, Beats per min.

Lastly, HR was affected by the interaction partners’ gender (χ^2^(2) = 11.26, *p* = 0.004), but gender IP also interacted with the participant’s gender (χ^2^(2) = 18.30, *p* < 0.001; Fig. 4A). Pairwise *t*-tests revealed higher HR in women when interacting with female versus male IP (*t*(1514) = 2.60, *p* = 0.005). In contrast, men showed lower HR when interacting with female versus mixed IP (*t*(1475) = − 2.83, *p* = 0.015) and tended to show lower HR when interacting with female versus male IP (*t*(1507) = − 2.13, *p* = 0.070). Comparing male and female participants, men showed lower HR than women when interacting with female IP (*t*(131) = − 5.44, *p* < 0.001).

### Heart rate variability

Details of the results for the RMSSD (i.e., HRV) model are presented in Table 2 (right column). VIF (all ≤ 7.45) were in an acceptable range^72^. In accordance with the state anxiety model, RMSSD was higher (i.e., more adaptive) when participants interacted with more familiar interaction partners (β = − 0.03, *p* < 0.001). In accordance with the HR results, RMSSD was higher during virtual versus direct social interactions (χ^2^(1) = 8.62, *p* = 0.003), and a marginal main effect for the participant’s gender (β = − 0.10, *p* = 0.071) indicated a tendency towards lower RMSSD in women. The participant’s gender also interacted with familiarity IP (χ^2^(1) = 25.99, *p* < 0.001; Fig. 3B). Simple slope analyses revealed a positive slope for women (β = 0.02, *p* = 0.004) but a negative slope for men (β = − 0.03, *p* < 0.001).

**Figure 5:**
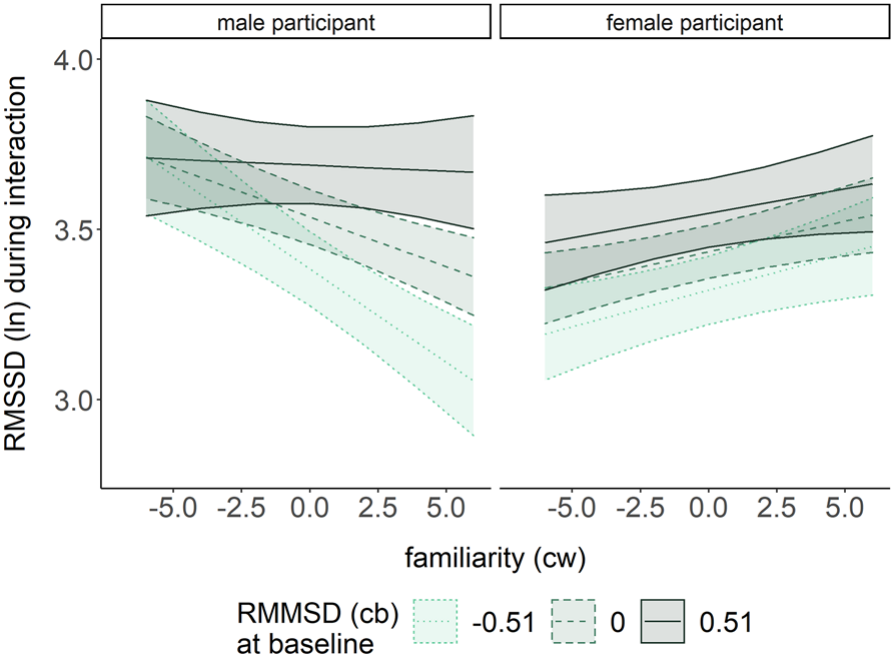
Prediction of RMSSD by familiarity in male (left panel) and female (right panel) participants at low (-1SD), medium, and high (+ 1SD) levels of baseline RMSSD. cb, Centred between; cw, Centred within.

Due to the main effects of autonomic baseline values and gender participant in the HR and RMSSD models, we additionally tested if baseline levels affected the gender participant x familiarity IP effects by testing the main models against a model with an additional three-way interaction (gender participant x familiarity IP x baseline). In the HR model, this interaction was neither significant (χ^2^(1) = 0.04, *p* = 0.833) nor improved model performance (*p* = 0.691). In the RMSSD model, the three-way interaction was significant (χ^2^(1) = 9.76, *p* = 0.002; see Fig. 5) and improved model fit (*p* = 0.005). Separate simple slope analyses in the male and female subsample revealed a negative slope for men with low baseline RMSSD levels (β = − 0.06, *p* = 0.010), indicating that the gender-specific negative relationship between familiarity and RMSSD in men was limited to those with low baseline RMSSD, while the positive relation in women was independent of baseline RMSSD.

Lastly, as for HR, gender IP interacted with the participant’s gender (χ^2^(2) = 9.54, *p* = 0.008; Fig. 4B). Pairwise *t*-tests revealed lower RMSSD in women when interacting with female versus male IP (*t*(1515) = − 0.11, *p* = 0.008). There were no other significant contrasts (all *p*s’ ≥ 0.220).

## Discussion

Overcoming limitations of previous social buffering studies in laboratory settings, this EMA study investigated how important characteristics of an interaction partner such as their familiarity and gender affect social buffering of state anxiety and related cardiovascular responses during real-life social interactions in women and men. Our results showed a decrease in state anxiety with increasing familiarity of the interaction partner (Fig. 2) which was independent of the gender of the participant and their interaction partner(s). In parallel, we assessed HR and HRV, i.e., cardiovascular responses related to subjective state anxiety ratings [see also^18,20^]. HR and HRV were more sensitive to gender differences than the subjective anxiety ratings. Specifically, our results showed a decrease in HR and an increase in HRV with increasing familiarity of the interaction partner in women but not in men (Fig. 3) and suggested opposite-gender social buffering effects on the autonomic level (Fig. 4).

The finding of reduced state anxiety with more familiar interaction partners in both men and women replicated the previously found social buffering effect of familiarity on subjective anxiety measures^32–34^ in everyday life. This effect was gender-independent. Extending previous findings^36,37^, we revealed that the social buffering effect of familiarity on autonomic anxiety-related responses differed between genders. The decrease in HR and the increase in HRV when women interacted with more familiar persons are in accordance with other findings of stronger social buffering effects in women^3,21^ and imply that women benefit more from the presence of a familiar interaction partner than men. The tend-and-befriend theory may provide explanations for this gender-dependent familiarity effect. This theory proposes that women have evolved to respond to stress by engaging in nurturing and protective behaviours towards social connections, seeking social support, and forming strong social bonds^73,74^. In the context of the present study, this suggests that women may engage in interactions with more familiar interaction partners for stress reduction, given that these persons are more likely to provide social support, and thus, to lead to stress and anxiety-reducing effects^5,33^. Empirical evidence further indicates that women reported greater tendencies towards tend-and-befriend and flight responses during stress, while men reported more fight responses^74^. In line with this, higher perceived social support under conditions of higher stress was previously associated with higher HRV in women but not in men. In turn, this aligns with previous findings which imply different stress management strategies in women versus men^75^. In our study, gender-dependent HRV levels suggest that men might have engaged in more adaptive autonomic regulation (i.e., higher HRV) when confronted with less familiar interaction partners. Previously, men but not women have shown adaptive HRV in negative social situations (e.g., negative social evaluation)^76^ as well as more adaptive stress responses after receiving social support from strangers^22^. As social interactions with less familiar interaction partners could be perceived as (more) negative^77^, this could translate to more adaptive responses in men and less autonomic emotion regulation in women during such situations. Unfamiliar interaction partners could therefore have affected women more negatively than men, similar to evidence of a higher vulnerability of women to stress-induced hyperarousal^18,78^ as well as their higher tendency towards tend-and-befriend or flight behaviours^73,74^.

In the present study, women also showed higher general levels of autonomic arousal than men, represented in higher HR during social interactions. Thus, in comparison to women, men’s need for social buffering may have been smaller as their cardiac arousal was already lower, leading to the lack of autonomic social buffering effects of familiarity in men. However, the exploratory finding of HRV-reducing effects of familiarity in men at lower (i.e., less adaptive) baseline HRV levels refutes this argument. The maladaptive effect of higher familiarity particularly emerged for those men more likely to require social buffering^12,13^, wheras even women with higher (i.e., more adaptive) baseline HRV profited from the social buffering effect of familiarity. These findings further support the novel gender-specific effect. Overall, the observed reductions of state anxiety scores and autonomic responses with increasing familiarity are in line with previous laboratory and EMA findings^34,36,37^. Extending these previous results, our findings show that in everyday life, the presence of a familiar interaction partner has a differential effect on cardiovascular responses of women versus men. The finding of differences in autonomic responses but not in subjective anxiety scores is in line with previous findings showing social buffering effects on the autonomic but not the subjective level^19,22,29^. It is possible that anxiety-altering effects of gender only emerged on the autonomic level because they were suppressed or misconceived on the subjective level. Factors like social norms could cause participants not to admit to gender-related differences in state anxiety^79,80^.

Evidence regarding gender effects in social buffering have been largely conflicting, ranging from stronger social buffering effects in women^3,21^, vice versa^22,23^, to no gender differences in social buffering^26,27^. Most previous studies primarily focused on the participant’s gender, while the gender of the interaction partner was either kept constant^3,22^ or was not included in the main analyses^23^. The present findings of autonomic opposite-gender effects are novel, showing that social buffering effects in everyday life are shaped by the gender of both the participant and their interaction partners. In greater detail, our results showed stronger autonomic social buffering effects if participants interacted with persons from the opposite gender (Fig. 4). In women, this was reflected in decreased HR and increased HRV in the presence of male compared to female interaction partners^28,29^. Similarly, men showed lower HR in the presence of female interaction partners compared to mixed-gender social interactions.

Previous studies investigating opposite-gender effects often focussed on romantic partners^22,28,81^. Importantly, entering romantic relationship as a control variable into our models neither improved model fit nor distinguished the opposite-gender social buffering effects, which rules out the assumption that these effects were mainly driven by the romantic partner. Men’s reduced HR when interacting with women agrees with other results on social buffering effects of a female interaction partner in men only^19,22,81^, while the stronger social buffering effect in women in the presence of a male interaction partner is in contrast to anxiety-buffering effects of female dyads found in the laboratory^3,19^. This could have different causes relating to methodology. For instance, conclusions on same versus opposite-gender social buffering effects from previous laboratory studies may be limited as many of them only investigated two-person interactions and did not compare all possible gender constellations, e.g., investigating same-gender^3^ or opposite-gender dyads^22^ but not both. Factors like role expectations^82^ could also be more salient and influential in laboratory compared to EMA settings and bias participants’ experience and behaviour, e.g., female participants wanting to fulfil expectations of getting along with other women and thus acting accordingly^83,84^.

With our EMA approach, we were able to assess everyday-life social interactions with a variety of male and female interaction partners (e.g., differing in quantity, familiarity). Extending previous studies, this allowed us to investigate the effects of social interactions with multiple interaction partners and in different gender constellations outside the laboratory, thus identifying gender-dependent social buffering effects in everyday-life social interactions. In contrast to other studies, our analyses did not only include present or very recent social interactions (e.g., within the previous five minutes)^12^, but also interactions that had ended anytime within the 30 min prior to the EMA prompt. In addition, we considered the length of the social interactions which could be as short as 1 min. Main effects in our models revealed that longer interactions related to lower state anxiety and higher HRV. Our findings thus suggest that social buffering effects on state anxiety may endure and last over several minutes (< 30 min) and that autonomic and subjective anxiety reductions—while being stronger after longer social interactions—can evolve even from brief contact. Note that men’s lower HR with female compared to mixed interaction partners hints towards higher anxiety-related responses during mixed-gender social interactions. This aligns with previous findings on such differential effects^85,86^, including stronger anxiety induction by same versus mixed-gender social partners^87,88^. By default, mixed-gender interactions included two or more interaction partners, whereas interactions with female or male interaction partners could also include just one person. Interestingly, our models revealed that more interaction partners were associated with lower HRV levels, implying that dyads relate to more adaptive autonomic responses than group interactions. Note that exploratory analyses showed that the opposite-gender effect did not depend on interaction partner quantity. Future research should look more closely into the diverse effects of same versus mixed-gender social interactions in everyday life.

The results of the current study show that EMA combining smartphones and wearable sensors is a useful and sensitive tool to investigate the effects of complex social interactions in everyday life. In the current study, we used this set-up in healthy and young participants with relatively low anxiety, most of whom were university students. To enhance the present findings’ generalizability, future studies should validate our findings with clinical (e.g., from the anxiety-disorder spectrum) as well as more diverse samples (e.g., differing in age and/or socioeconomic and educational background). Moreover, other potential influences on cardiovascular activity such as hormonal fluctuations in relation to women’s menstrual cycle or use of oral contraceptives^89,90^ could be considered. Finally, our participants interacted more frequently with familiar versus unfamiliar interaction partners. A study including more social interactions with unfamiliar interaction partners could confirm the stability of the familiarity effects. Such a study should also include a baseline measurement of state anxiety to investigate whether familiar or opposite-gender interaction partners reduce anxiety levels beyond baseline.

In summary, the present EMA study provides novel insights into gender-dependent changes in anxiety-related autonomic responses during social contacts. Based on our results, social contacts with more familiar male interaction partners could be best suited to help reduce anxiety-related autonomic responses in women. At the same time, female interaction partners (or practitioners) may be best suited for anxiety reductions in men, while their familiarity might be disregarded. In conclusion, our study underlines the importance of considering gender in the context of social buffering.

## Supplementary Information

Supplementary Information.

## Data Availability

The data and analyses presented in this study can be found in the following online repository: https://osf.io/d3tg5/?view_only=0679d2a299254ac0a0d60672c8ad6975.
