# Supplementary material for: The effects of everyday-life social interactions on anxiety-related autonomic responses differ between men and women
Source: Sci Rep. 2023 Jun 12;13:9498. doi: 10.1038/s41598-023-36118-z (PMC10261044; doi:10.1038/s41598-023-36118-z)
Supplement: Supplementary file 1 — Supplementary Information. [file 41598_2023_36118_MOESM1_ESM.pdf]

Supplementary Material:

The effects of everyday-life social interactions on anxiety-related autonomic responses differ between men and women

Marthe Gründahl, Martin Weiß, Kilian Stenzel, Jürgen Deckert, & Grit Hein

Methods

Additional clinical questionnaires

Additional exploratory questionnaires were the German version of the Emotion Regulation Questionnaire [ERQ; 1], the Multidimensional Scale of Perceived Social Support [MSPSS; 2], the NEO Five Factor Inventory for the assessment of personality dimensions [3,4] and, due to the context of the Covid-19 pandemic and related social distancing measures, the Loneliness and Isolation during Social Distancing (LISD) Scale [5]. All questionnaires were presented on a computer (no missing values allowed). The two subscales of the 10-item ERQ assess cognitive reappraisal and expressive suppression and are rated on a Likert scale from 1 ("strongly disagree") to 7 ["strongly agree"; 1,6]. The 12-item MSPSS measures perceived adequacy of social support from family, friends, and significant others rated on a Likert scale from 0 ("strongly disagree) to 5 ["strongly agree"; 2]. The 60-item NEO-FFI assesses the five personality dimensions Extraversion (E), Neuroticism (N), Agreeableness (A), Conscientiousness (C), and Openness for Experience (O). Each is assessed by twelve items rated on a 5-point Likert scale ranging from "strongly agree" to "strongly disagree" [3,4]. The LISD scale measures state and trait aspects of loneliness and isolation that are associated with deprivations in mental health, e.g., state anxiety. Its two state factors (lonely and isolated, connected and supported) are measured in the context of social distancing, accompanied by three trait factors (loneliness and isolation, sociability and sense of belonging, social closeness and support). Items are rated on a 5-point Likert scale from "strongly agree" to "strongly disagree" [5].

Complete list of EMA survey items

EMA surveys were delivered with the movisensXS app (Movisens GmbH, Karlsruhe, Germany) on an android study smartphone. For interactions ≤ 30 min ago, participants answered the social interaction questionnaire. The wording of the survey was adjusted to the interaction's time (present/past) and interaction partner quantity and gender. For interactions > 30 min ago, they answered an alternative questionnaire with a similar structure on their

current activity to obviate reactivity (i.e., false answers in order to receive a shorter questionnaire). Detailed item lists are presented in Supplementary Tables S1 (social interaction questionnaire) and S2 (alternative questionnaire; see S3 and S4 for German items). Each questionnaire started with an introductory sentence: “Thank you for taking the time to answer our questions. Please read everything carefully and click on the answer that applies most.” (German: „Vielen Dank, dass du dir Zeit nimmst, um unsere Fragen zu beantworten. Bitte lies dir alles sorgfältig durch und klicke auf die Antwort, die am ehesten zutrifft“.). After indicating the time of their latest social interaction, participants were asked to answer the subsequent questions in regard to their latest social interaction (“now”, “within the last 30 min”) or current situation and activity (“more than 30 min ago”) with the following phrase: “Please answer the following questions in regard to this social interaction/your current activity.” (German: “Bitte beantworte die folgenden Fragen in Bezug auf diese soziale Interaktion/deine momentane Situation und Tätigkeit.”).

Supplementary Tables S1 and S2 present the social interaction questionnaire and the alternative activity questionnaire in English, respectively, while Supplementary Tables S3 and S4 present the social interaction questionnaire and the activity questionnaire in German, respectively.

Supplementary Table S1

Items of the social interaction questionnaire translated into English.

Item	Question	Response option	Reference
1.1 - 1.10	10-item short form of the STAI's state anxiety subscale	Likert scale: 1 (not at all), 8 (totally)	[7]
2	When did your latest social interaction take place?	- now<br>- within the last 30 min<br>- more than 30 min ago	
3	The interaction is/was predominantly…	- private<br>- job-related	
4	Type of social interaction:	- direct contact<br>- telephone call<br>- e-mail/letter<br>- SMS<br>- social media	
4.1	(if Item 4 = "social media")<br>Type of Social Media:	- social network (app / website)<br>- chat / messenger<br>- video / voice-chat<br>- others	
4.2	(if Item 4.1: "social network")<br>Type of communication	- created post<br>- commented on post(s)<br>- commented on photo(s)<br>- commented on video(s)<br>- others	
5.1	When did the interaction start?	Visual scale, three anchors (left, middle, right):<br>< 1 Min, 15 Min, > 30 Min	
5.2	How long is the interaction presumably going to last? / How long did the interaction last?	Visual scale, three anchors (left, middle, right):<br>< 1 Min, 15 Min, > 30 Min	
6	With how many people are you interacting with/did you interact with?	Likert scale: 1, 2, 3, 4, at least 5	
7.1	Interaction partner (multiple choice possible):	- partner<br>- family<br>- friend<br>- colleague<br>- acquaintance<br>- stranger	[8],[9]
7.2	Gender of this person/ these persons:	- female<br>- male<br>- mixed	
	Please rate the following statements about the social interaction.		
8.1	I know the other person [one of the other persons] well.	Likert scale: 1 (not at all), 8 (very)	[9],[10]
8.2	The other person [one of the other persons] is similar to me.	Likert scale: 1 (not at all), 8 (very)	[11]
8.3	I am close to the other person [one of the other	Likert scale: 1 (not at all), 8 (very)	[9],[10]

Item	Question	Response option	Reference
	persons].		
	I worry/worried about …		
9.1	… what the other person[s] thought of me.	Likert scale: 1 (not at all), 9 (very)	[12],[13]
9.2	… that I could/would say or do the wrong things.	Likert scale: 1 (not at all), 9 (very)	
10	How (un)pleasant is/was the interaction?	Likert scale: 1 (very unpleasant), 8 (very pleasant)	[14]
11	Which of the following substances did you consume within the last hour? (multiple choices possible)	- solid food<br>- stimulating / caffeinated drink<br>- nicotine<br>- analgesic<br>- alcohol<br>- other mind-altering substance<br>- none	

Note. Square brackets and slashes indicate alternative wording, depending on the time (present, past) of the social interaction and the number of interaction partners.

Supplementary Table 2

Items of the alternative activity questionnaire translated into English.

Item	Question	Response option
1.1 - 1.10	10-item short form of the STAI's state anxiety subscale	Likert scale: 1 (not at all), 8 (totally)
2	When did your latest social interaction take place?	- now<br>- within the last 30 min<br>- more than 30 min ago
3	The activity is predominantly…	- private<br>- job-related
4	(if item 3 = "private")<br>current activity:	- sport<br>- locomotion<br>- reading/watching TV<br>- getting information/news<br>- entertainment/playing games<br>- planning/organizing<br>- eating/drinking<br>- relaxing<br>- daily tasks, e.g., household<br>- others
4	(if item 3 = "job-related")<br>current activity:	- planning/organizing<br>- learning/practicing/educating myself<br>- collecting information/reading<br>- working on a computer<br>- physical labour<br>- manual work/handicraft<br>- creative working/development<br>- eating/drinking<br>- relaxing/taking a break<br>- others

Item	Question	Response option
5.1	When did the activity start?	Visual scale, three anchors (left, middle, right):<br>< 1 Min, 15 Min, > 30 Min
5.2	How long is the activity presumably going to last?	Visual scale, three anchors (left, middle, right):<br>< 1 Min, 15 Min, > 30 Min
6	How many people are near you?	Likert scale: 1, 2, 3, 4, at least 5
7.1	(if item 6 ≠ “0”)<br>Persons near you: (multiple choices possible)<br><br>(if item 6 = “0”)<br>Who was last near you? (multiple choices possible)	- partner<br>- family<br>- friend<br>- colleague<br>- acquaintance<br>- stranger
7.2	Gender of this person/these persons:	- female<br>- male<br>- mixed
	Please rate the following statements about the current activity.	
8.1	I know the current activity well.	Likert scale: 1 (not at all), 8 (very)
8.2	The current activity is arduous.	Likert scale: 1 (not at all), 8 (very)
8.3	The current activity is important to me.	Likert scale: 1 (not at all), 8 (very)
9	How (un)pleasant is the activity?	Likert scale: 1 (very unpleasant), 8 (very pleasant)
10	Which of the following substances did you consume within the last hour?<br>(multiple choices possible)	- solid food<br>- stimulating / caffeinated drink<br>- nicotine<br>- analgesic<br>- alcohol<br>- other mind-altering substance<br>- none

Supplementary Table S3

Items of the social interaction questionnaire in German.

Item	Question	Response option
1.1 – 1.10	10-Item Kurzform der STAI State-Angst Subskala	Likert scale: 1 (überhaupt nicht zutreffend), 8 (sehr zutreffend)
2	Wann war deine letzte soziale Interaktion?	- aktuell<br>- vor max. 30 Min.<br>- vor mehr als 30 Min
3	Die Interaktion ist/war vorwiegend…	- privat<br>- beruflich
4	Art der sozialen Interaktion:	- Persönlicher Kontakt<br>- Telefonat<br>- E-Mail/Brief<br>- SMS<br>- Social Media
4.1	(if Item 4 = „Social Media”)<br>Art von Social Media:	- Soziales Netzwerk (App / Website)<br>- Chat / Messenger<br>- Video / Voice-Chat<br>- Sonstige
4.2	(if Item 4.1: „Soziales Netzwerk“)<br>Art der Kommunikation:	- Post verfasst<br>- Post(s) kommentiert<br>- Foto(s) kommentiert<br>- Video(s) kommentiert<br>- Sonstige
5.1	Wann begann die Interaktion?	Visual scale, three anchors (left, middle, right):<br>< 1 Min, 15 Min, > 30 Min
5.2	Wie lange wird die Interaktion vermutlich noch dauern? / Wie lange dauerte die Interaktion?	Visual scale, three anchors (left, middle, right):<br>< 1 Min, 15 Min, > 30 Min
6	Mit wie vielen Personen interagierst du / hast du interagiert?	Likert scale: 1, 2, 3, 4, mind. 5
7.1	Interaktionspartner*innen (Mehrfachauswahl möglich):	- Partner*in<br>- Familie<br>- Freund*in<br>- Kolleg*in<br>- Bekannte*r<br>- Fremde*r
7.2	Geschlecht dieser Person(en):	- weiblich<br>- männlich<br>- gemischt
	Bitte bewerte die folgenden Aussagen zur Interaktion.	
8.1	Ich kenne die andere Person [eine beteiligte Person] gut.	Likert scale: 1 (überhaupt nicht), 8 (sehr)
8.2	Die andere Person [eine beteiligte Person] ist mir ähnlich.	Likert scale: 1 (überhaupt nicht), 8 (sehr)

Item	Question	Response option
8.3	Die andere Person [eine beteiligte Person] steht mir nah.	Likert scale: 1 (überhaupt nicht), 8 (sehr)
	Ich mache mir Sorgen darüber / habe mir Sorgen darüber gemacht, …	
9.1	… was die andere[n] Person[en] über mich denkt / denken.	Likert scale: 1 (überhaupt nicht), 9 (sehr)
9.2	… dass ich etwas Falsches sagen oder machen könnte.	Likert scale: 1 (überhaupt nicht), 9 (sehr)
10	Wie (un-)angenehm ist / war die Interaktion?	Likert scale: 1 (sehr unangenehm), 8 (sehr angenehm)
11	Welche der folgenden Substanzen hast du innerhalb der letzten Stunde konsumiert? (Mehrfachauswahl möglich)	- feste Nahrung<br>- Anregendes / koffeinhaltiges Getränk<br>- Nikotin<br>- Analgetikum<br>- Alkohol<br>- andere bewusstseins-verändernde Substanz<br>- Keine

Note. Square brackets and slashes indicate alternative wording, depending on the time (present, past) of the social interaction and the number of interaction partners

Supplementary Table 4

Items of the alternative activity questionnaire in German.

Item	Question	Response option
1.1 – 1.10	10-Item Kurzform der STAI State-Angst Subskala	Likert scale: 1 (überhaupt nicht zutreffend), 8 (sehr zutreffend)
2	Wann war deine letzte soziale Interaktion?	- aktuell<br>- vor max. 30 Min.<br>- vor mehr als 30 Min
3	Die Tätigkeit ist vorwiegend…	- privat<br>- beruflich
4	(if item 3 = „privat“)<br>Aktuelle Tätigkeit:	- Sport<br>- Fortbewegung<br>- Lesen / Fernsehen<br>- Informieren / Nachrichten<br>- Unterhaltung / Spielen<br>- Planen / Organisieren<br>- Essen / Trinken<br>- Entspannen<br>- Alltägliche Aufgaben, z.B. Haushalt<br>- Sonstiges
4	(if item 3 = „beruflich“)<br>Aktuelle Tätigkeit:	- Planen / Organisieren<br>- Lernen / Üben / Fortbilden<br>- Informationen sammeln / Lesen<br>- Am PC arbeiten<br>- Körperliche Arbeit<br>- Feine Arbeit / Handarbeit<br>- Kreative Arbeit / Entwicklung<br>- Essen / Trinken

Item	Question	Response option
		- Entspannen / Pause<br>- Sonstiges
5.1	Wann begann die aktuelle Tätigkeit?	Visual scale, three anchors (left, middle, right):<br>< 1 Min, 15 Min, > 30 Min
5.2	Wie lange wird die Tätigkeit vermutlich noch dauern?	Visual scale, three anchors (left, middle, right):<br>< 1 Min, 15 Min, > 30 Min
6	Wie viele Personen befinden sich in deiner Nähe?	Likert scale: 1, 2, 3, 4, mind. 5
7.1	(if item 6 ≠ „0“)<br>Personen in deiner Nähe: (Mehrfachauswahl möglich)<br><br>(if item 6 = „0“)<br>Wer war zuletzt in deiner Nähe? (Mehrfachauswahl möglich)	- Partner*in<br>- Familie<br>- Freund*in<br>- Kolleg*in<br>- Bekannte*r<br>- Fremde*r
7.2	Geschlecht dieser Person(en):	- weiblich<br>- männlich<br>- gemischt
	Bitte bewerte die folgenden Aussagen zur aktuellen Tätigkeit.	
8.1	Ich kenne die aktuelle Tätigkeit gut.	Likert scale: 1 (überhaupt nicht), 8 (sehr)
8.2	Die aktuelle Tätigkeit ist anstrengend.	Likert scale: 1 (überhaupt nicht), 8 (sehr)
8.3	Die aktuelle Tätigkeit ist mir wichtig.	Likert scale: 1 (überhaupt nicht), 8 (sehr)
9	Wie (un-)angenehm ist die aktuelle Tätigkeit?	Likert scale: 1 (sehr unangenehm), 8 (sehr angenehm)
10	Welche der folgenden Substanzen hast du innerhalb der letzten Stunde konsumiert? (Mehrfachauswahl möglich)	- feste Nahrung<br>- Anregendes / koffeinhaltiges Getränk<br>- Nikotin<br>- Analgetikum<br>- Alkohol<br>- andere bewusstseinsverändernde Substanz<br>- Keine

ECG data curation and heart rate variability calculation

A more detailed description of the ECG data curation and HRV calculation with the movisens DataAnalyzer software is presented below. Prior to data processing, we visually inspected the ECG data. We excluded nine participants (7.5%) at this stage due to frequent artefacts mainly caused by insufficient quality of chest belts that were later replaced. We applied the movisens DataAnalyzer software (version 1.13.8) to the remaining 111 data sets to convert the ECG signals into HRV indices. The software uses an automated algorithm to detect artefacts. The DataAnalyzer algorithms' output is based on minute-by-minute calculations derived from 2-minute segments, which were averaged every 60 s. The procedure for the detection of R-peaks was adapted from [15]. The detection of artefacts was based on the raw ECG signal. Signal amplitude and number of zero crossings per seconds outside a normal physiological range were marked as artefacts. The filter for R-peaks and artefacts exclusion is based on recommendations by [16]. This filter checked for valid changes of consecutive RR-intervals and R-peak amplitudes, and removed interbeat intervals outside the range of 250-2000 ms, as well as R-peak amplitudes outside the range of 0.1-5.0 mV, producing an NN-list. The maximum variation was set at 20% for the RR intervals and at 30% for the R amplitudes. The RR interval lists contains 2-minute segments of 30-second-shifts. Segments with an insufficient number of NN intervals for further analysis were excluded. To achieve stationarity criteria of the HR for subsequent HRV calculation, the NN intervals were detrended [17]. The raw acceleration data was averaged over 30-s periods, from which mean acceleration per 60-s epoch was derived. ECG and EMA data were aligned using the movisens DataMerger software.

Additional statistical analysis

All statistical analyses were conducted in R (version 4.2.0). We calculated Pearson correlations between HR and RMSSD at baseline, HR and RMSSD during EMA (mean per participant), and clinical questionnaires (SIAS, ADS-K). In addition to sociodemographic data and clinical questionnaires reported in the main manuscript, we computed means and

standard deviations for additional clinical questionnaires. Mean values of sample characteristics between men and women were compared using *t*-tests.

Additional Exploratory Results

Social interaction characteristics

Table S5 presents additional characteristics of the 1 536 social interactions assessed by the EMA surveys.

Supplementary Table S5

Social interaction characteristics of the total sample (N = 96).

	M	*SD*	*Min*	*Max*	*n*
Familiarity IP	6.28	2.18	1	8	1536
Pleasantness SI	6.17	1.46	1	8	1536
SI duration (in s)	1197.23	679.07	60	3000	1536
SI duration (in min)	19.95	11.32	1	50	1536

Note. Table contains non-centred values. IP = interaction partner. SI = social interaction.

Correlations between trait questionnaires and autonomic responses

Correlations between HR, RMSSD, and clinical questionnaires are presented in Table S6. Mean levels of HR and RMSSD showed negative relations at BL and during EMA. Depressive symptoms correlated positively with mean HR during EMA and with trait social interaction anxiety (for numeric values, see Table S6).

Supplementary Table S6

*Correlations between heart rate, heart rate variability (RMSSD) and clinical questionnaires for the total sample (*N *= 96).*

	RMSSD (BL)	HR (EMA)[1]	RMSSD (EMA)[1]	SIAS	ADS-K
HR (BL)	-.59***	.62***	-.24*	-.03	.12
RMSSD (BL)		-.21*	.47***	.15	.13
HR (EMA)[1]			-.54***	.07	.22*
RMSSD (EMA)[1]				-.06	-.09
SIAS					.32**

Note. [1]mean per participant. ADS-K = Allgemeine Depressionsskala (engl.: Center for Epidemiologic Studies Depression Scale); BL = baseline; EMA = ecological momentary assessment; HR = heart rate; RMSSD = the root mean square of successive differences between heartbeats; SIAS = Social Interaction Anxiety Scale.
* $p < .05$, ** $p < .01$, *** $p < .001$.

Sample characteristics

Numeric values for additional sample characteristics and gender differences are reported in Table S7. *t*-tests revealed that compared to female participants, male participants showed lower dispositional use of cognitive reappraisal and higher use of emotion suppression. Regarding personality dimensions, results showed higher O and a tendency towards lower C in men compared to women. Trait sociability and sense of belonging tended to be lower in female vs. male participants (for numeric Values, see Table S7).

Supplementary Table S7

Characteristics of the total sample and the female and male participants.

	Total sample (N = 96)		Female subsample (N = 51)		Male subsample (N = 45)		Sample comparison	*p*-value
	M	*SD*	*M*	*SD*	*M*	*SD*		
CR[1]	26.03	6.97	27.37	6.67	24.51	7.06	$t(94) = -2.04$	.044
ES[1]	13.74	5.02	12.73	5.16	14.89	4.66	$t(94) = 2.13$	.035
MSPSS	67.72	8.94	68.53	8.66	66.80	9.25	$t(94) = -0.94$	.347
Extraversion[2]	38.11	5.91	37.94	6.16	38.13	5.67	$t(94) = 0.30$	.761
Neuroticism[2]	24.91	9.18	25.57	9.50	24.16	8.86	$t(94) = -0.75$	.455
Agreeableness[2]	37.54	7.00	38.29	6.30	36.69	7.71	$t(94) = -1.12$	.265
Conscientiousness[2]	41.38	8.29	43.12	8.36	39.40	7.83	$t(94) = 1.86$	.066
Openness[2]	37.14	6.23	36.04	6.34	38.38	5.93	$t(94) = -2.24$	.028
State Loneliness[3]	2.70	0.86	2.80	0.82	2.59	0.90	$t(94) = -1.20$	.232
State Support[3]	3.65	1.38	3.44	1.49	3.89	1.20	$t(94) = 1.59$	.115
Trait Loneliness[3]	2.43	1.21	2.60	1.31	2.24	1.06	$t(93.36) = -1.48$	.142
Trait Sociability[3]	3.48	1.08	3.29	1.12	3.70	0.99	$t(94) = 1.88$	.064
Trait Support[3]	3.64	1.34	3.49	1.46	3.82	1.19	$t(93.34) = 1.24$	.216

Note. [1] Subscales from the Emotion Regulation Questionnaire (ERQ); [2] Subscales from the NEO Five Factor Inventory (NEO-FFI) indicating personality dimensions; [3] Subscales from the Loneliness and Isolation during Social Distancing (LISD) Scale. CR = Cognitive Reappraisal; ES = Expressive Suppression; MSPSS = total score of the Multidimensional Scale of Perceived Social Support; Openness = Openness for Experience; State Loneliness = lonely and supported; State Support = connected and supported; Trait Loneliness = loneliness and isolation; Trait Sociability = sociability and sense of belonging; Trait Support = social support and closeness.

References

1. Abler, B. & Kessler, H. Emotion Regulation Questionnaire - Eine deutschsprachige Fassung des ERQ von Gross & John. *Diagnostica* **55**, 144–152 (2009).
2. Zimet, G.D., Dahlem, N.W., Zimet, S.G. & Farley, G.K. The Multidimensional Scale of Perceived Social Support. *Journal of Personality Assessment* **52**, 30-41 (1988).
3. Costa, P.T. & McCrae, R.R. *Revised NEO Personality Inventory (NEO-PI-R) and NEO Five-Factor Inventory (NEO-FFI) professional manual*, (Psychological Assessment Resources, Odessa, FL, 1992).
4. Borkenau, P. & Ostendorf, F. *NEO-FFI: NEO-Fünf-Faktoren Inventar nach Costa und McCrae, Manual*, (Hogrefe, Göttingen, 2008).
5. Gründahl, M. et al. Construction and validation of a scale to measure loneliness and isolation during social distancing and its effect on mental health. *Frontiers in psychiatry* **13**(2022).
6. Gross, J.J. & John, O.P. Individual differences in two emotion regulation processes: implications for affect, relationships, and well-being. *J Pers Soc Psychol* **85**, 348-62 (2003).
7. Grimm, J. State-Trait-Anxiety Inventory nach Spielberger. Deutsche Lang-und Kurzversion. *Methodenforum der Universität Wien: MF-Working Paper* (2009).
8. Hur, J. et al. Social context and the real-world consequences of social anxiety. *Psychological Medicine* **50**, 1989-2000 (2019).
9. Venaglia, R.B. & Lemay Jr, E.P. Hedonic Benefits of Close and Distant Interaction Partners: The Mediating Roles of Social Approval and Authenticity. *Personality and Social Psychology Bulletin* **43**, 1255-1267 (2017).
10. Vogel, N., Ram, N., Conroy, D.E., Pincus, A.L. & Gerstorf, D. How the social ecology and social situation shape individuals' affect valence and arousal. *Emotion* **17**, 509-527 (2017).

11. Qi, Y.Y. et al. The mere physical presence of another person reduces human autonomic responses to aversive sounds. *Proceedings of the Royal Society B-Biological Sciences* **287**, 20192241 (2020).

12. Goodman, F.R., Stiksma, M.C. & Kashdan, T.B. Social Anxiety and the Quality of Everyday Social Interactions: The Moderating Influence of Alcohol Consumption. *Behavior therapy* **49**, 373-387 (2018).

13. Kashdan, T.B. et al. A contextual approach to experiential avoidance and social anxiety: evidence from an experimental interaction and daily interactions of people with social anxiety disorder. *Emotion* **14**, 769-781 (2014).

14. Cornelius, T., Birk, J.L., Edmondson, D. & Schwartz, J.E. The joint influence of emotional reactivity and social interaction quality on cardiovascular responses to daily social interactions in working adults. *J Psychosom Res* **108**, 70-77 (2018).

15. Hamilton, P. Open source ECG analysis. in *Computers in cardiology* 101-104 (IEEE, Memphis, TN, USA, 2002).

16. Clifford, G., McSharry, P. & Tarassenko, L. Characterizing artefact in the normal human 24-hour RR time series to aid identification and artificial replication of circadian variations in human beat to beat heart rate using a simple threshold. in *Computers in cardiology* (Memphis, TN, USA, pp. 129-132, 2002).

17. Eleuteri, A., Fisher, A.C., Groves, D. & Dewhurst, C.J. An Efficient Time-Varying Filter for Detrending and Bandwidth Limiting the Heart Rate Variability Tachogram without Resampling: MATLAB Open-Source Code and Internet Web-Based Implementation. *Computational and Mathematical Methods in Medicine* **2012**(2012).
